# Inactivation of Polyomavirus SV40 as Surrogate for Human Papillomaviruses by Chemical Disinfectants

**DOI:** 10.3390/v13112207

**Published:** 2021-11-02

**Authors:** Martin Hufbauer, Ulrike Wieland, Jürgen Gebel, Jochen Steinmann, Baki Akgül, Maren Eggers

**Affiliations:** 1 Institute of Virology, National Reference Center for Papilloma- and Polyomaviruses, University of Cologne, Faculty of Medicine and University Hospital of Cologne, 50935 Cologne, Germany; Ulrike.wieland@uk-koeln.de (U.W.); baki.akguel@uk-koeln.de (B.A.); 2 Institute for Hygiene and Public Health, University Hospital Bonn, 53127 Bonn, Germany; juergen.gebel@ukb.uni-bonn.de; 3 Dr. Brill + Partner GmbH Institute for Hygiene and Microbiology, 28259 Bremen, Germany; jochen-steinmann@web.de; 4 Labor Prof. Dr. G. Enders MVZ GbR, 70193 Stuttgart, Germany; eggers@labor-enders.de

**Keywords:** human papillomavirus (HPV), HPV16 pseudovirus, simian virus 40 (SV40), disinfection, quantitative suspension test

## Abstract

Human papillomaviruses (HPV) are non-enveloped DNA viruses infecting cutaneous and mucosal squamous epithelia. Sexually transmitted HPV-types that are carcinogenic to humans such as HPV16 can induce cervical and other anogenital cancers. Virus transmission through fomites such as inadequately disinfected gynecological equipment is a further potential transmission route. Since HPV cannot be easily grown in cell culture, polyomavirus SV40 has been used as a surrogate virus when testing the virucidal activity of chemical disinfectants. So far, studies that have compared the virucidal activity of different disinfectants against HPV and SV40 are lacking. Here, we evaluated the susceptibility of HPV16 pseudovirus and SV40 to seven active biocidal substances using quantitative suspension tests. Ethanol, glutaraldehyde (GTA), dodecyldipropylentriamin (DPTA), and ortho-phthalaldehydes (OPA) were able to reduce the infectivity of HPV16 pseudovirus >99.99% after 5 min. In contrast, isopropanol, peracetic acid (PAA), and quaternary ammonium compounds with alkylamines (QAC) only led to a slight or no reduction in infectivity. Concerning SV40, only GTA (60 min contact time), PAA, and OPA had virus-inactivating effects. In conclusion, the virucidal activity of three out of seven disinfectants tested was different for HPV16 pseudovirus and SV40. In this study, SV40 was shown to be a reliable surrogate virus for HPV when testing isopropanol-, GTA-, QAC-, and OPA-based disinfectants.

## 1. Introduction

Anogenital human papillomaviruses (HPV) are transmitted by sexual contact and HPV in risk group 1, which are carcinogenic to humans, can cause high-grade cervical, vaginal, vulvar, and anal intraepithelial neoplasia and the respective anogenital cancers [1,2,3]. However, HPV can also be transmitted by non-sexual means such as surface contaminations and inappropriately sanitized gynecological ultrasound equipment. Thus, infection control and prevention are of paramount importance with respect to delivering safe and high-quality care to patients undergoing procedures such as vaginal sonography [4,5,6,7,8]. Several studies have shown that intracavity ultrasound probes can be contaminated with HPV-DNA, and viral DNA can also be found throughout the environment of gynecological examination rooms [9,10]. If automated chemo-thermal disinfection is not applicable because of the sensitivity of the instruments, it is of particular interest to use an evaluated and effective disinfectant for manual reprocessing with short exposure times. It should therefore be mandatory using chemical disinfectants with proven efficacy against HPV when disinfecting such medical devices. 

The HPV life cycle is tightly linked to the host cell differentiation, which makes it difficult to grow HPV in standard cell cultures. To overcome this difficulty, different cell culture systems to produce recombinant viral particles have been developed. So called quasiviruses have a capsid consisting of codon optimized L1 and L2 proteins and contain an HPV genome. Pseudoviruses also have codon optimized L1 and L2 capsid proteins but, in contrast to quasiviruses, encapsidate a GFP reporter plasmid (pseudogenome) that can easily be tracked microscopically in cellular infectivity assays [11,12,13,14,15].

In the past, both HPV and the polyomavirus simian virus 40 (SV40) were assigned to the family Papovaviridae, a now abolished family, which was split into the two families Papillomaviridae and Polyomaviridae in 1999 [16]. Based on the fact that HPV, in contrast to the polyomavirus SV40, cannot be propagated in monolayer cell cultures, SV40 has been used in Germany since 1982 [17] as a surrogate virus in tests of chemical disinfectants regarding their effectiveness to inactivate HPV virions. Comparative studies on the inactivation of HPV and SV40 by disinfectants have not yet been performed. In the present study, we comparatively evaluated the susceptibility of HPV16 pseudovirus and SV40 to different disinfectants using quantitative suspension tests.

## 2. Materials and Methods

### 2.1. Chemical Disinfectants, Conditions, and Parameters Used in This Study

In order to prevent intra-laboratory variations, calibrated solutions of disinfectants used in the HPV16 pseudovirus and SV40 assays were provided by the task force for interlaboratory surveys (ring trials) of the Association of Applied Hygiene (VAH) c/o Institute for Hygiene and Public Health, University Hospital Bonn, Bonn, Germany (https://vah-online.de/en/dmk-working-groups, (accessed on 5 June 2021)). The substances tested are listed in Table 1 and Table 2.

Ethanol and isopropanol were included due to their widespread use in surface disinfectants and hand sanitizers. Glutataldehyde (GTA) and quaternary ammonium compounds (QAC) with alkylamines are commonly used as disinfectants in clinical settings, and peracetic acid (PAA), dodecyldipropylentriamin (DPTA), and orthophtalic acid (OPA) are used for the disinfection of medical instruments and surfaces.

### 2.2. HPV16 Pseudovirus Production and Titration

**Figure 1:**
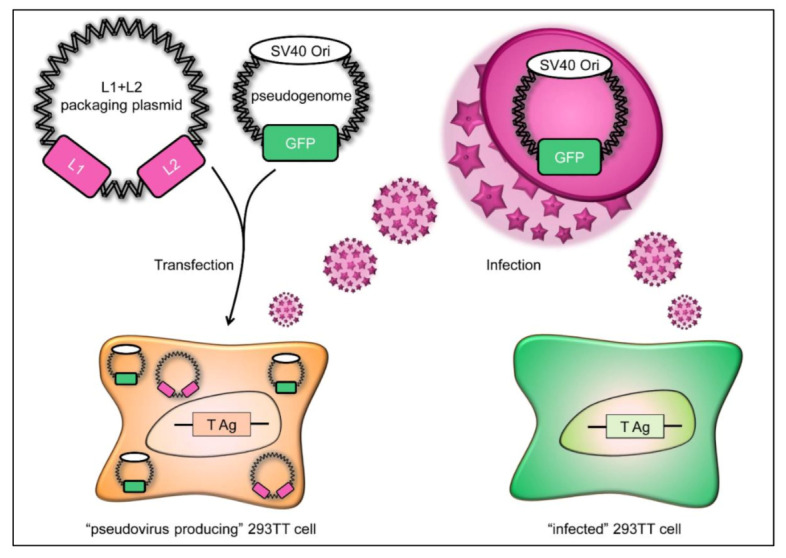
Schematic diagram of HPV16 pseudovirus production. The packaging plasmid encoding the L1 and L2 capsid proteins (pink) is transfected into 293TT cells along with a pseudogenome encoding the green fluorescent protein (GFP; shown in green), resulting in “pseudovirus-producing” 293TT cells. The pseudogenome carries a SV40 origin of replication (SV40 Ori), allowing high expression in 293TT cells. These cells synthesize L1 and L2 capsids that assemble into virus particles carrying the pseudogenome. These pseudoviruses can then be used to infect 293TT cells, which in turn express GFP encoded by the pseudogenome, which can then be visualized under a microscope, as shown in Figure 2.

The human embryonic kidney cell line 293TT was maintained in Dulbecco’s modified Eagle’s medium (DMEM) (Life Technologies, Carlsbad, CA, USA) with 10% fetal calf serum (FCS) (Life Technologies), 1× penicillin-streptomycin (10,000 units/mL penicillin and 10,000 units/mL streptomycin) (Life Technologies) and supplemented with 250 µg/mL hygromycin B (Thermo Fisher Scientific, Waltham, MA, USA). Cell cultures were incubated at 37 °C in a humidified 6% CO_2_ atmosphere. 293TT cells replicate plasmids with the SV40 origin of replication to a very high copy number, because they express the SV40 T-antigens in their nucleus, enabling replication of the HPV16 pseudovirus genome (Figure 1) [11].

**Figure 2:**
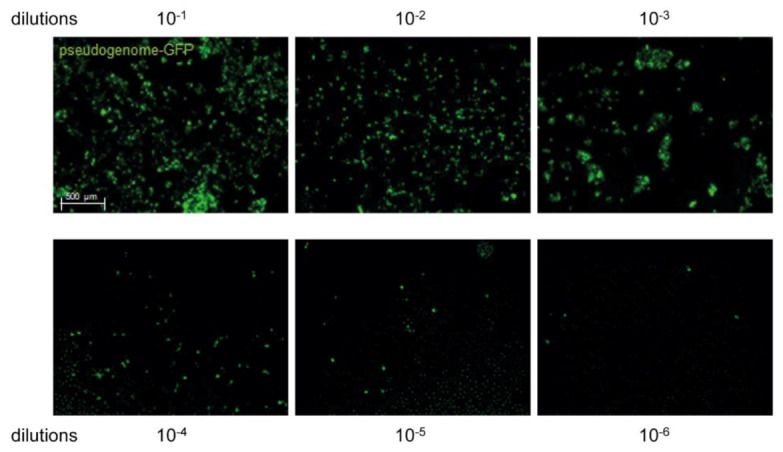
Representative images of HPV16 pseudovirus-infected 293TT cells. Cells were incubated with 10^−1^ to 10^−6^ dilutions of HPV16 pseudovirus and cultivated for 48 h. 293TT cells expressing HPV16 pseudogenome-GFP appear in green.

Detailed technical protocols and nucleotide maps of plasmids used in this work have been described elsewhere [12]. Briefly, 293TT cells were transfected with an HPV16-L1/L2 expression plasmid, p16sheLL [18], and an EGFP-expressing plasmid pCIneoEGFP [13], which carries a pseudogenome with a SV40 origin, using Lipofectamine 2000 (Life Technologies, Carlsbad, CA, USA), according to the manufacturer’s protocol. After 48 h, transfected cells were harvested and resuspended in PBS at very high density (>100 million cells/mL). After the addition of 1/20th volume of 10% Triton X-100, 1/1000th volume of RNase mix (Ambion cat# 2286) and 1/40th volume of 1 M ammonium sulfate (pH 9), the lysate was incubated at 37 °C overnight. After 24 h of maturation, lysates were washed and clarified by centrifugation at 5000× *g* for 5 min. The final wash step was conducted with PBS containing 0.8 M NaCl. Pseudovirus containing supernatants were loaded onto an Optiprep (Sigma-Aldrich, Steinheim, Germany) gradient (27%, 33%, and 39%) and centrifuged for 3.5 h at 16 °C at 50,000 rpm (234,000× *g*) in an SW55ti rotor. Virus titers were determined by incubating 293TT cells (1 × 10^4^ cells/well in 96-well plates) with a 1:10 dilution series of HPV16-pseudovirus fractions (n = 8 repeats for each dilution). After 48 h, cells were analyzed for GFP expression by fluorescence microscopy (Figure 2) using a Leica DMI 6000B microscope equipped with a Leica DFC365 FX camera and analyzed with Leica LAS X imaging software (v3.3.0.16799). Virus titers were calculated by the Spearman and Kärber method [19,20].

### 2.3. Quantitative Suspension Test Using HPV16 Pseudovirus

**Figure 3:**
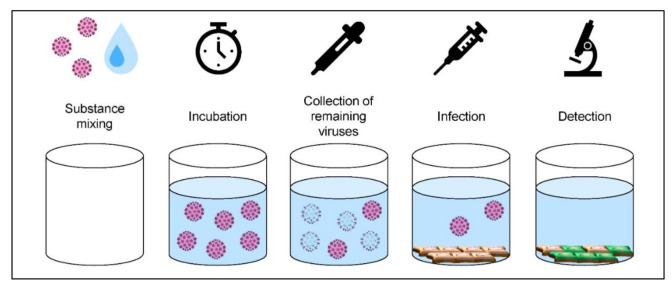
Quantitative suspension test using HPV16 pseudovirus. First, HPV16 pseudovirus (titer of at least 10^8^ tissue culture infectious dose, 50% (TCID_50_/mL) and BSA (final concentration in the test procedure 0.3 g/L BSA) were mixed in equal volumes. This solution was then exposed to 8 volumes of the respective disinfectant (Table 1) and incubated at 20 °C ± 2 °C for the contact time indicated in Table 2. At the end of the contact time, cell culture media were added to the solution to stop the reaction. For determination of the virus titer after incubation with the respective disinfectants, 293TT cells (1 × 10^4^ cells/well in 96-well plates) were incubated with 10^−1^ to 10^−10^ dilution series of HPV16 pseudovirus/disinfectant solution (n = 8 repeats for each dilution). Cells were analyzed after 48 h for GFP expression by fluorescence microscopy.

Quantitative suspension tests were performed according to the Guidelines of the German Association for the Control of Virus Diseases (DVV)/Robert Koch Institute (RKI) [21]. The interfering substance (bovine serum albumin (BSA, Thermo Fisher Scientific)) was used according to EN 14476:2015 [22]. A schematic overview of the procedure is shown in Figure 3.

The remaining virus titer was again calculated using the Spearman and Kärber method [19,20]. The RF was calculated as the difference between the remaining HPV16 pseudovirus titer after exposure to disinfectant and unexposed pseudovirus. Cytotoxicity of the disinfectants was determined by serially tenfold dilution of the respective disinfectants in sterile water (Ampuwa, Fresenius Kabi) and subsequent incubation of 293TT cells (37 °C and 6% CO_2_ for 48 h).

### 2.4. Production of SV40 Test Virus Suspension

The SV40 stock virus suspension was produced according to the DVV/RKI Guidelines. Polyomavirus Simian virus 40 (SV40) strain 777 and CV-1 cells, an immortalized rhesus monkey kidney cell line for virus cultivation and suspension tests, were kindly provided by the DVV virus bank (Prof. Dr. A. Sauerbrei of the former Institute for Virology and Antiviral Therapy of the University Hospital Jena). CV-1 cells were cultivated at 37 °C in a humidified 5% CO_2_ atmosphere. The cells were fed with Dulbecco’s minimum essential medium (D-MEM) supplemented with heat-inactivated 10% FCS and non-essential amino acids. For virus cultivation, confluent monolayers with a maximum age of two days were used and infected with SV40. Cells showing a cytopathic effect were harvested and virus was isolated by one freeze and thaw cycle. Cell debris was separated by low-speed centrifugation at 400× *g* for 10 min. Aliquots of the virus suspension were stored at −70 °C.

### 2.5. Quantitative Suspension Tests with SV40

Tests were performed in two of our laboratories (Stuttgart and Bremen) according to the Guidelines of the German Association for the Control of Virus Diseases (DVV)/Robert Koch Institute (RKI) [21]. The interfering substance (BSA) was used according to EN 14476:2015 [22]. One part by volume of SV40 virus suspension (titer of at least 10^7^−10^8^ tissue culture infectious dose, 50% (TCID_50_/mL)) and one part by volume of the interfering substance (final concentration 0.3 g/L BSA) was mixed with eight parts by volume of the disinfectant at 20 °C ± 2 °C. Infectivity was determined in microtiter plates by means of end point dilution titration. At the end of the chosen contact time (Table 2), the reaction was immediately stopped by serial dilutions with ice-cold cell culture medium. In six to eight wells of a sterile polystyrene flat-bottomed 96-well microtiter plate containing a confluent monolayer of CV-1 cells (10–15 × 10^3^ cells per well), 100 μL from each dilution was placed. Cultures were observed for cytopathic effects (CPE) for at least 14 and up to 21 days after exposure. All tests were conducted in three independent test runs that were performed on different days in two independent laboratories, respectively. Virus controls were evaluated after the longest contact time. The RF was calculated as the difference between the remaining SV40 virus titer after exposure to disinfectant and unexposed SV40. Cytotoxicity was determined by serial tenfold dilution with sterile water of standardized hardness instead of test virus suspension.

### 2.6. Statistical Analysis

The virus titers were determined using the Spearman and Kärber method [19,20] and expressed as TCID_50_/mL with standard deviation. Titer reduction is presented as the difference between the virus titer after contact time with the disinfectant and the titer of unexposed virus. This difference is given as titer reduction factor ((RF), log_10_ reduction of viral titer) with standard deviation. A reduction in viral titers of ≥4 log_10_ steps (inactivation ≥ 99.99%) was regarded as proof of virucidal activity.

## 3. Results

In this study, we evaluated the susceptibility of HPV16 pseudovirus and SV40 to different disinfectants in order to determine whether SV40 is an appropriate surrogate virus for HPV in the context of studies on chemical disinfectants. To this end, we compared HPV16 pseudovirus and SV40 with regard to their susceptibility to ethanol, isopropanol, and five further disinfectants (Table 1 and Table 2).

### 3.1. Efficacy of Chemical Disinfectants against HPV16 Pseudovirus

**Figure 4:**
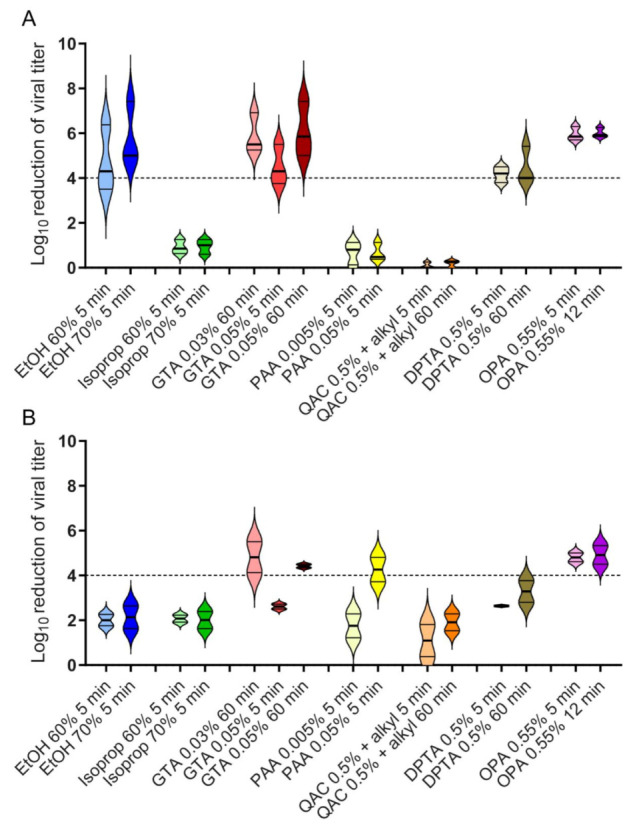
Activity of chemical disinfectants against HPV16 pseudovirus and SV40. The results from quantitative suspension tests with (**A**) HPV16 pseudovirus and (**B**) SV40 and chemical disinfectants are shown as the reduction factor (RF). Tests were performed at 20 °C ± 2 °C with 0.3 g/L BSA as an interfering substance. The data are an average of n = 3 experiments for HPV16 pseudovirus conducted in one laboratory (Cologne). In the case of SV40, n = 6 experiments were conducted as three independent experiments in two different laboratories (Stuttgart and Bremen). The error bars show the standard deviation. The dashed line indicates a ≥4 log_10_ reduction in virus titer after different contact times, corresponding to a minimum of 99.99% inactivation. Alkyl: alkylamines.

Experiments with the HPV16 pseudovirus showed that longer contact times (12 min and 60 min) did not have a statistically significant impact on infectivity compared to 5 min (Figure 4A). From the alcohols tested, only ethanol at both concentrations analyzed (60% and 70%) led to a decrease in infectivity >99.99%, while isopropanol (60% and 70%) showed no virucidal activity. If a disinfectant reduces infectivity >99.99%, which corresponds to titer reduction by ≥4 log_10_ steps, it is defined as virucidal activity. Disinfectants based on GTA, DPTA, and OPA were also able to reduce virus titers by >99.99% in all concentrations and contact times tested (Table 2, Figure 4). On the other hand, disinfectants based on PAA and QAC with alkylamines only resulted in a nonsignificant very slight (PAA) decrease in infectivity or had no effect (QAC) on infectivity (Table 2, Figure 4A).

### 3.2. Efficacy of Chemical Disinfectants against Polyomavirus SV40

In parallel to the experiments with HPV16 pseudovirus, comparative quantitative suspension experiments were performed with SV40. Ethanol, isopropanol, and disinfectants based on QAC with alkylamines and DPTA reduced the infectivity by only approximately two log_10_ steps under all conditions tested and thus were not considered to exhibit virucidal activity. The same was true for GTA 0.05% and PAA 0.005% at a contact time of 5 min, respectively. However, SV40 incubated for 60 min with disinfectants based on 0.03% or 0.05% GTA were shown to reduce infectivity ≥4 log_10_ steps. The same was true for 0.05% PAA (contact time 5 min) and 0.55% OPA (contact time 5 min and 12 min) (Table 2, Figure 4B).

### 3.3. Comparison of the Effectiveness of Chemical Disinfectants against HPV16 Pseudovirus and SV40

Of the disinfectants tested, only GTA 0.03% and 0.05% at a contact time of 60 min and OPA 0.55% at contact times of 5 and 12 min, respectively, showed virucidal activity against both HPV16 pseudovirus and SV40. Ethanol (60% and 70%, 5 min), DPTA 0.5% (5 min and 60 min), and GTA 0.05% at a contact time of 5 min were only effective against HPV16 pseudovirus, while PAA 0.05% at a contact time of 5 min only inactivated SV40. Isopropanol (60% and 70%, 5 min), PAA 0.005% (5 min), and QAC 0.5% (5 min and 60 min) were ineffective against both HPV16 pseudovirus and SV40 (Table 2, Figure 4A,B).

## 4. Discussion

Up until now, European Guidelines lack a suitable test virus to validate the efficacy of disinfectants against HPV [22]. In contrast, Germany has a long tradition of testing SV40 as a reference virus for papillomaviruses [21,23,24,25]. However, there is still a degree of uncertainty as to whether SV40 is a suitable surrogate for virus inactivation studies that meets the European Standards. In the present work, we investigated the virus-inactivating properties of several chemical disinfectants against the HPV16 pseudovirus and SV40. 

In a previous study by Meyers et al. (2014), HPV16 native viruses, isolated from organotypic culture systems and quasivirus were analyzed for their resistance against chemical disinfectants. The authors tested ethanol and isopropanol and even after a contact time of 45 min, no reduction in infectivity of both virus stocks was observed with both 70% and 95% ethanol [26]. In their study, 70% isopropanol with a contact time of 45 min was effective against quasivirions, but not against HPV16 native virions [26]. Regarding these two alcohols, we obtained the opposite results to Meyers et al. (2014). We detected a virucidal effect of both 60% and 70% ethanol against HPV16 pseudovirus at a contact time of only 5 min. Furthermore, we did not observe a significant effect of 60% and 70% isopropanol at a contact time of 5 min on thee infectivity of both HPV16 pseudovirus and SV40. The longer contact time applied for isopropanol and the use of different types of recombinant virus particles for which minor structural differences still cannot be excluded [14] may explain the contradictory results to Meyers et al.. Although we and Egawa et al. (2021) tested pseudoviruses against ethanol, different effects were observed in both studies. Whether the use of GFP-based (this study) or luciferase-based (Egawa et al. (2021)) pseudogenomes are responsible for these differences needs to be tested in parallel in future studies.

In addition to ethanol, we also detected a strong reduction in HPV16 pseudovirus titer after incubation with disinfectants based on GTA, DPTA, or OPA. The virucidal activity of OPA found in our study is in accordance with Egawa et al. (2021), who have recently shown that OPA inactivates HPV16 pseudovirus and HPV18 native virions [27]. In the study by Meyers et al. (2016), OPA was not effective against HPV16 and HPV18 native viruses, pointing also at differences in the preparation of native viruses used in different studies [28]. As far as PAA-based disinfectants are concerned, we did not observe an effect on the HPV16-pseudovirus titer. This result is consistent with the findings of Meyers et al. (2014), who also did not observe a significant virucidal activity of 0.25% PAA-silver on HPV16 quasi- and native-virus [26]. Our data with SV40 revealed no activity of ethanol and isopropanol against SV40, which is in line with previously published data [25]. In accordance with the literature, we demonstrated that PAA-based disinfectants are active against SV40 at a concentration of 0.05% [25]. For disinfectants based on QAC + alkylamines, we did not observe a significant reduction in SV40 titers, which is in contrast to a previous study [25]. As QAC are a heterogeneous group of molecules, this discrepancy might be explained with the use of different QAC based solutions. Furthermore, an about three log_10_ reduction in SV40 virus titer was measured with disinfectants based on DPTA (contact time 60 min). This indicates that SV40 could be susceptible to this substance. However, the predefined requirement for virucidal activity was not met by DPTA for SV40. Interestingly, significant virucidal activity against SV40 was observed for disinfectants based on OPA (contact time 5 min) and GTA (contact time 60 min). 

When comparing the results for HPV16 pseudovirus and SV40, we found that both were resistant against 60% or 70% isopropanol and 0.5% QAC+ alkylamines. In contrast, 60% and 70% ethanol could inactivate HPV16 pseudovirus after a contact time of 5 min, but not SV40. With respect to ethanol-based disinfectants, SV40, being more resistant to ethanol, can be seen to cover HPV16. The use of SV40 as a surrogate for HPV16 in the case of PAA needs to be seen with caution as we could show that 0.05% PAA is active only against SV40, but not against HPV16 pseudovirus. Here, either a more resistant virus (e.g., parvovirus [29] or HPV16 pseudovirus) should be tested. Interestingly, both test viruses were inactivated by disinfectants based on GTA (contact time 60 min) and OPA, in the latter case, already after 5 min. Taken together, SV40 can be used as a surrogate for HPV with some but not all disinfectants. Therefore, extra care should be taken depending on the disinfectant used.

In summary, the use of SV40 as a surrogate virus for HPV is suitable when testing isopropanol-, GTA-, QAC-, and OPA-based disinfectants. Although ethanol- and DPTA-based disinfectants were not active against SV40, they showed virucidal activity against the HPV16 pseudovirus and therefore might be used as disinfectants. Thus, the virucidal activity of three out of seven active biocidal substances tested in our study was different for the HPV16 pseudovirus and SV40. Of the four biocidal substances with concordant results, isopropanol and QAC were ineffective and OPA was effective against both viruses. GTA was effective for both viruses only after an incubation time of 60 min. However, whether a contact time of 60 min for disinfection of gynecological instruments with GTA is feasible in examination rooms needs to be determined. Future comparative studies should further evaluate the virucidal activity of disinfectants against HPV using the different HPV-based test systems (native virions, quasivirus, and pseudovirus) to clarify the partly contradictory results of published studies.

## Figures and Tables

**Table 1:** Chemical disinfectants tested.

Active Biocidal Substance	Company
Ethanol (EtOH), ≥99.5%	Roth (Karlsruhe, Germany)
Isopropanol (Isoprop.), ≥99.8%	Roth (Karlsruhe, Germany)
Glutaraldehyde (GTA)	Dow (Midland, MI, USA)
Peracetic Acid (PAA)	Stockmeier (Bielefeld, Germany)
Quaternary Ammonium Compounds (QAC) + alkylamines	B.Braun (Melsungen, Germany)
Dodecyldipropylentriamin (DPTA)	Lonza (Basel, Switzerland)
Orthophthalic Acid (OPA)	Advanced Sterilization Products (Irvine, CA, USA)

**Table 2:** Comparison of virucidal activity of chemical disinfectants against HPV16 pseudovirus and SV40. The selection was made based on the availability of the products on the market and the known concentrations of their active ingredients. The active substances of the used disinfectants, the concentration, the contact time, and the reduction factor (RF) values are listed. The RF (log_10_ reduction of viral titer) was calculated as the difference between the remaining virus titer after exposure to disinfectant and unexposed virus. Shown is the average RF of three (HPV pseudoviruses) or six (SV40) independent experiments with standard deviation, respectively. Green background indicates a ≥4 log_10_ reduction of virus titer corresponding to a viral inactivation of >99.99%, red background indicates a RF < 4 log_10_. Tests were performed at 20 °C ± 2 °C with 0.3 g/L BSA as the interfering substance.

Active Substance	Concentration	Contact Time	RF + SD HPV Pseudovirus	RF +SD SV40
EtOH	60%	5 min	4.73 ± 1.49	2.00 ± 0.36
	70%	5 min	5.81 ± 1.40	2.14 ± 0.71
Isoprop.	60%	5 min	0.91 ± 0.31	2.08 ± 0.22
	70%	5 min	0.95 ± 0.33	2.01 ± 0.54
GTA	0.03%	60 min	5.89 ± 0.90	4.82 ± 0.97
	0.05%	5 min	4.52 ± 0.90	2.61 ± 0.16
	0.05%	60 min	6.10 ± 1.23	4.42 ± 0.11
PAA	0.005%	5 min	0.69 ± 0.51	1.76 ± 0.76
	0.05%	5 min	0.66 ± 0.41	4.26 ± 0.76
QAC + alkylamines	0.5%	5 min	0.08 ± 0.14	1.10 ± 1.01
	0.5%	60 min	0.18 ± 0.16	1.92 ± 0.53
DPTA	0.5%	5 min	4.17 ± 0.35	2.64 ± 0.04
	0.5%	60 min	4.47 ± 0.82	3.29 ± 0.69
OPA	0.55%	5 min	5.95 ± 0.31	4.81 ± 0.28
	0.55%	12 min	6.00 ± 0.21	4.92 ± 0.59

## Data Availability

Not applicable.
